# High Fat Diet-Induced CD8^+^ T Cells in Adipose Tissue Mediate Macrophages to Sustain Low-Grade Chronic Inflammation

**DOI:** 10.3389/fimmu.2021.680944

**Published:** 2021-06-23

**Authors:** Sonia Kiran, Vijay Kumar, E. Angela Murphy, Reilly T. Enos, Udai P. Singh

**Affiliations:** ^1^ Department of Pharmaceutical Sciences, College of Pharmacy, The University of Tennessee Health Science Center, Memphis, TN, United States; ^2^ Pathology, Microbiology, and Immunology, School of Medicine, University of South Carolina, Columbia, SC, United States

**Keywords:** obesity, inflammation, chemokine, CD8^+^ T cells, macrophages

## Abstract

Obesity in the United States and worldwide reached epidemic proportions within the last 20 years. Obesity is a very powerful health determinant or indicator that facilitates the development and progression of several metabolic diseases, insulin resistance, and low-grade chronic inflammation. Low-grade chronic inflammation in adipose tissue (AT) is marked by the accumulation of T cells, macrophages, and other immune cells and increased production of proinflammatory cytokines. During the onset of obesity but before the influx of macrophages, the AT is infiltrated by T cells that are strongly implicated in the initiation of obesity-associated inflammation. In comparing mice fed a high-fat diet (HFD) with those fed a normal diet (ND), we observed in HFD epididymal AT induction and infiltration of activated T cells, an accumulation and polarization of macrophages, and an increase in populations of activated CD4^+^ T cells and CD8^+^ T cells that express CXCR3 or killer cell lectin-like receptor subfamily G member 1 (KLRG1). Levels of inflammatory cytokines and leptin and the results of *in vitro* co-culture experiments revealed interactions among HFD- and ND-induced CD8^+^ T cells, macrophages, and adipocytes. Our findings suggest that obese tissues activate and induce both CD4^+^ and CD8^+^ CD69^+^ T cells and augment the expression of CXCR3 receptors, which promotes the recruitment and numbers of pro-inflammatory M1 macrophages to maintain low-grade chronic inflammation. The results support the hypothesis that CXCR3-expressing CD8^+^T cells play an essential role in the initiation and maintenance of adipose tissue inflammation.

**Graphical Abstract:**
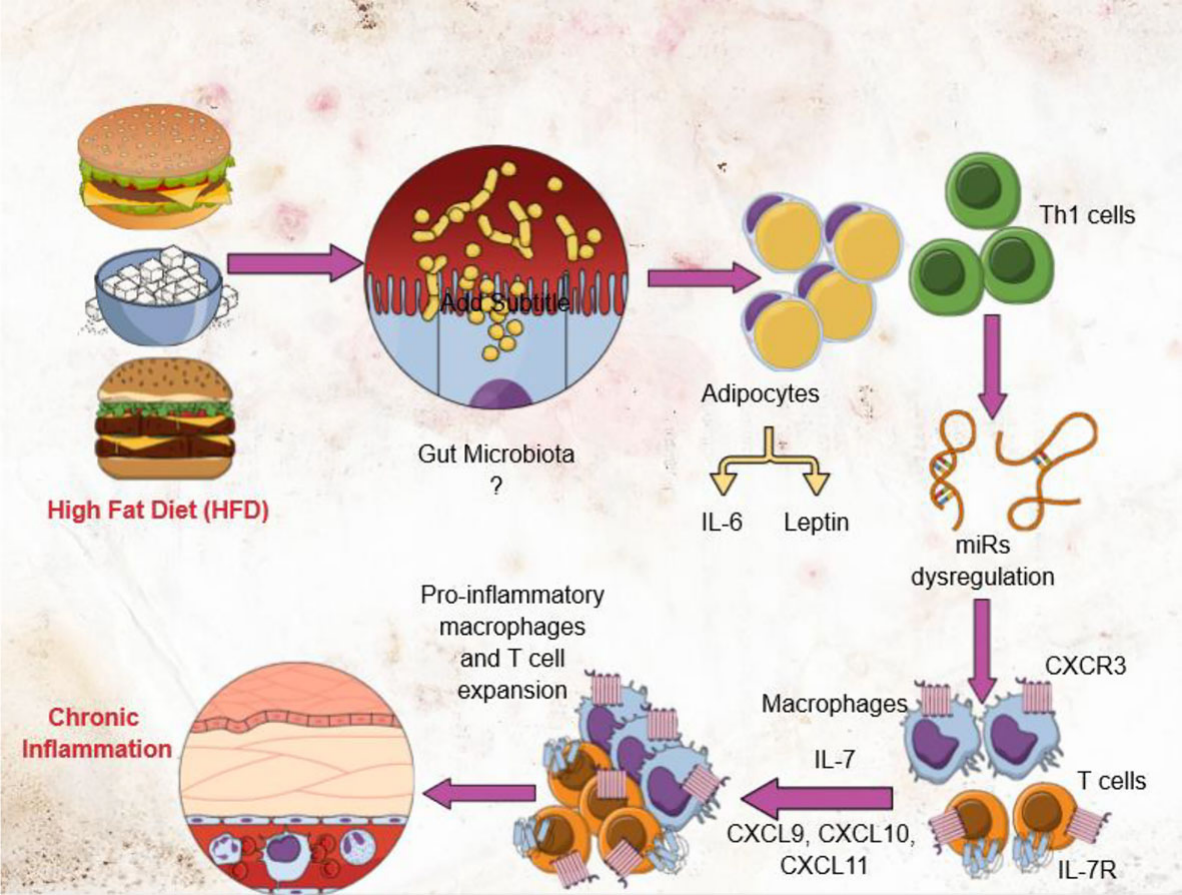


## Introduction

Obesity has reached pandemic proportions and affects virtually all age, socioeconomic groups of people around the world. Currently, more than half of the American population is obese or overweight, with women being disproportionately affected (1, 2). Epidemiological studies indicate that consumption of a high-fat diet (HFD) is well correlated with the incidence of obesity. One hallmark of obesity and metabolic disease is low-grade chronic inflammation in adipose tissue (AT) marked by the accumulation of T cells, macrophages, and other immune cells, and by an increase in the levels of proinflammatory cytokines (3). Obesity-induced inflammation is also crucial to the development of metabolic syndrome (MS), a constellation of conditions that includes hypertension, type 2 diabetes mellitus (T2DM), insulin resistance (IR), and atherosclerosis (4). The molecular basis for the relationship between obesity, chronic inflammation, and MS remains to be established. Resetting the immunological balance in obesity could represent an innovative approach for the management of MS. However, the early triggers and signals that sustain AT inflammation in obesity remain elusive, limiting our ability to effectively intervene to prevent and or suppress obesity.

T cells (primarily CD8^+^) play specific functions in obese AT (5, 6). Infiltration of T cells into AT followed by macrophage influx has been strongly implicated in the initiation of obesity-associated inflammation, since macrophages and CD8^+^ T cells interact with one another and regulate inflammation (7–9). Macrophages can mediate protective (anti-inflammatory, M2) and tissue destructive (pro-inflammatory) immune responses (10). While macrophages normally infiltrate the AT in both humans and mice, during obesity the total number of macrophages increases, and the ratio of M1 to M2 macrophages is altered (4, 11). In lean mice, the predominant macrophages in AT exhibit the M2 phenotype, utilize oxidative metabolism to maintain AT homeostasis and secrete anti-inflammatory cytokines (12). In obese mice, the number of M1 macrophages in AT increases, and the mice exhibit insulin resistance and inflammation (13). Effector CD8^+^ T cells (T_eff_) recruit macrophages during AT inflammation by a mechanism that remains unclear (9). Therefore, determining the underlying mechanism by which T_eff_ cells alter macrophage phenotype and function will provide crucial new knowledge that may be used to mitigate obesity.

The T helper-1 (Th1) biased CXCR3 chemokine receptor and its ligands CXCL9 and CXCL10 facilitate T cell differentiation, induce production of both memory and effector T cells, and maintain the balance between these two T cell populations (14, 15). CXCR3 is important for the proper functioning of T cells, NK cells, mast cells, and NKT cells (16, 17). The CXCR3 ligands CXCL9 and CXCL10 primarily bind to activated Th1 T cells, which express high levels of CXCR3 receptors (18). Adipocytes also express CXCR3 and modulate obesity-induced inflammation (19). We observed a significant increase in CXCR3^+^ T cells in mouse AT during high fat diet-induced obesity (DIO). Interestingly, treatment of LDL-receptor knockout mice with the CXCR3 antagonist NBI-74330 reduces both developments of, and accumulation of T_eff_ cells and macrophages in, atherosclerotic lesions and the relevant draining lymph nodes (20). The mechanism by which CXCR3 mediates the expansion of CD8^+^ T cells in AT inflammation is unclear. In the present study, we used a mouse model of HFD-induced obesity to study the relationship(s) between adipocytes, both CD4^+^, CD8^+^ T cells, CXCR3 expression, and macrophage function in the obese AT microenvironment. Our results showed that mice fed HFD exhibit increased expression of CXCR3 expressing CD8^+^ T cells and IFN-γ expressing CD4^+^ T cells which recruit and induce the number of macrophages their polarization towards M1, Th1 response, resulting in sustained low-grade chronic inflammation during obesity in mice.

## Materials and Methods

### Animals

Wild-type 6 to 8-week-old male C57BL/6 mice were purchased from Jackson Laboratories (Bar Harbor, ME). The mice were housed in the Animal Facility at the University of South Carolina School of Medicine from 2016 to 2018 for the initial two experiments. The mice were also housed in the Animal Facility at the University of Tennessee Health Science Center (UTHSC) from 2020 till date for further experiments. All mice were housed under humidity- and temperature-controlled conventional conditions in isolator cages with normal 12:12 hr light/dark cycles for a week for acclimatization to the animal facility. All animal experimentation was performed under protocols approved by the University of South Carolina’s Institutional Animal Care and Use Committee (IACUC) as well as from UTHSC (protocol number 20-0162) for handling and experimental procedures were performed to minimize pain and discomfort. Experimental groups consisted of 6 mice each and studies were repeated 3 times to obtain statistical relevance. Power analysis indicated that 6 mice per group will allow detection of effect sizes of 1.4 standard deviations between groups at a significance level of 5% and power of 80% using two-sided t-tests, conducted separately or within the context of ANOVA.

### High-Fat Diet-Induced Obesity in Mice

The composition of one of the most commonly used diets for inducing obesity in mice includes protein (20% Kcal), carbohydrates (20% Kcal), and fat (44% Kcal). ND contains 5% fat while AIN-76A-modified HFD contains 6% saturated and 34% unsaturated fat (both from Bio-Serv, Flemington NJ). Male 8-week-old C57BL/6 mice were fed with HFD (45% Kcal from fat) or normal diet (ND) for 16 weeks. The mice were monitored daily for general health and stool consistency to screen for the onset of diarrhea or other symptoms, weekly for blood glucose (Bayer glucometer, New Jersey), and twice a week for body weight. Dual-energy x-ray absorptiometry (Dexa) was done for each mouse separately every two weeks and at the time before euthanizing the mice. At the experimental endpoint (16 weeks after the onset of HFD and ND feeding), the animals were euthanized with an overdose of isoflurane. The spleen, mesenteric lymph nodes (MLNs), and epididymal fat tissues were collected for single-cell suspension as described below.

### Cell Isolation

At the DIO experimental endpoint, blood was collected and epididymal fat depots, MLNs, and spleens were harvested and processed for single-cell isolation as follows. Spleens and MLNs from individual mice of all groups were dissociated and spleen red blood cells (RBCs) were removed by incubation in lysis buffer (Sigma St. Louis, MO) and gentle centrifugation. Cell debris was further removed by passing the cell-containing supernatants through a sterile filter (Sigma St. Louis, MO). The resulting single-cell suspensions were washed twice with RPMI 1640 medium (Sigma St. Louis, MO), resuspended in the same medium supplemented with 10% fetal bovine serum (FBS), and placed on ice or at 4°C before their use in experimental procedures later on the same day.

Epididymal AT was collected from peripheral fat pads and gently placed in MACS C tubes (MACS Miltenyi Biotec, 130-096-334) containing digestion medium (Hanks balanced salt solution (HBSS), 2 mg/ml collagenase IV (Sigma-Aldrich), 2% FBS), and homogenized using a MACS dissociator. Following incubation for 30 min at 37°C with shaking, the cell suspension was passed through a 100-μm filter and centrifuged at 1200 rpm for 10 min to separate the stromal vascular-fraction (SVF) pellet from adipocytes in the supernatant. Collagenase digestion was repeated until adipocyte fractions were free of adherent cells to ensure recovery of the majority of the SVF population. Lymphocytes and adipocytes were maintained in a complete medium, as we described previously (21, 22). Serum concentrations of T helper cell-derived cytokines IL-6, TNF-α, MCP1 (monocyte chemoattractant protein-1), RANTES (regulated on activation cells expressed and presumably secreted), IL-1β, and CXCL10 were determined using a Luminex-based multiplex ELISA assay (Bio-Rad, Hercules, USA). Assay buffer containing a multiplex pool of fluorescent color-coded analyte beads conjugated to antibodies specific for mouse cytokines IL-6, TNF-α, MCP1, RANTES, IL-1β, or CXCL10 were added to pre-wet filter-bottom wells and 50 µl of assay beads. After removal of the buffer and a wash step, 50 µl of standard or mouse serum sample was added to each well and the plate was incubated for 1 hour with continuous gentle shaking (setting #3 of a Lab-Line™ Instrument Titer Plate Shaker, Melrose, IL). The plates were washed and vortexed for 30 seconds at 300 x g, 25 µl of biotinylated anti-mouse detection antibodies (Abs) specific for separate epitopes on the same cytokines were added to each well and the plates were incubated at room temperature for 30 minutes. 50 µl of the streptavidin-phycoerythrin (streptavidin-PE) detection solution was added and the plate was incubated with continuous shaking for 10 minutes at RT. Finally, 125µl of assay buffer was added and signals were detected with a Bio-Plex instrument using Luminex™ xMAP technology (Bio-Rad Laboratories, Austin, TX). Data analysis was performed using Bio-Plex Manager software (BioRad). This BioRad™ xMAP assay can detect >10 pg/ml for each analyte.

### Serum Leptin ELISA

Serum leptin levels were measured by an ELISA assay (Crystal Chem, Chicago, IL). The wells were washed with 300 µl of wash solution, 50µl of guinea pig anti-mouse leptin antiserum and 45 µl of sample diluent were added to each well, followed by 5µl of a serum sample or a reference standard in parallel wells. The plates were covered, incubated overnight at 4°C, then washed with assay solution. Anti-guinea pig IgG enzyme conjugate solution (100µl) was added and the plates were incubated at 4°C for 3 hours, then washed with buffer. 100µl of enzyme-substrate was added to each well and incubated at room temperature for 30 minutes. 100µl of stop buffer was added to each well and the serum leptin levels signals were detected at a wavelength of 450 nm using a microplate reader.

### Histology

The epididymal AT was washed with phosphate-buffered saline (PBS), cut longitudinally, fixed using 10% buffer neutral formalin followed by 4% paraformaldehyde, and embedded in paraffin. Fixed tissues were sectioned at 6 µm and stained with hematoxylin and eosin (H&E) for examination by microscopy. AT sections were examined for evidence of infiltration by immune cells by a blinded investigator.

### Co-Culture of HFD-and ND-Induced CD8^+^ T Cells, Adipocytes, and Circulating Monocytes

CD8^+^ T-cells and adipocytes from epididymal fat were isolated from HFD- and ND-fed mice using antibodies coupled to magnetic beads (Miltenyi Biotech). CD8^+^ T cells were further purified to >95% purity by cell sorter (FACS Aria II). Adipocyte obtained from HFD or ND fed mice were cultured alone or co-cultured with HFD- or ND-induced CD8**^+^** T cells and sorted purified circulating peripheral monocytes (CD11b^+high^ > 96% pure), that had been stimulated with anti-CD28 (1µg/ml) and anti-CD3 (5µg/ml) antibodies (BD PharMingen) and incubated at 37°C in 5% CO_2_. Following a 6-day incubation period, the cells were washed and stained with anti-CD11b and anti-F4/80 (macrophage marker) antibodies and analyzed by flow cytometry. Levels of CXCL10 and MCP1 that were secreted into the culture supernatant medium were analyzed by ELISA.

### Statistics

Several response variables were measured for each experimental unit within the two groups (HFD and ND) and the standard error of the sample mean was calculated. Values are shown ±SEM. T-tests within the context of ANOVA were used to show whether any differences in mean cytokine levels between groups were statistically significant. If the usual normality assumptions were not valid, data were transformed to the natural log scale or nonparametric methods including Friedman’s test, the Kruskal-Wallis test, and the Wilcoxon-Mann-Whitney rank-sum test was used. SAS software (SAS Institute, Inc., Cary NC) was used for statistical analysis. Values were considered significant when p-values were ≤0.05 (5% level of significance).

## Results

### High-Fat Diet Induces Alteration in Metabolic Parameters and Body Weight

**Figure 1:**
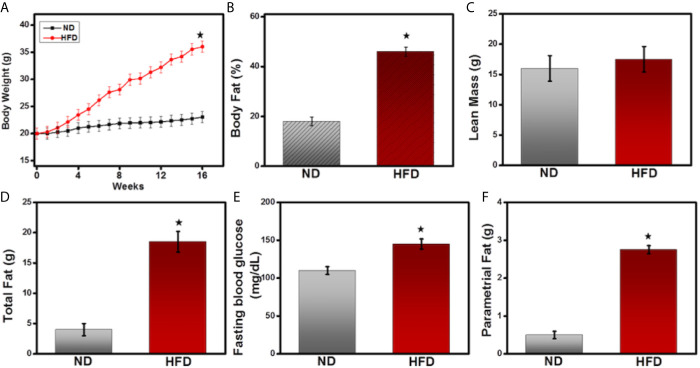
High-fat diet induces metabolic parameters and body weight. 8-week-old male C57BL6 mice were fed a normal diet (ND, □) or a high-fat diet (HFD, ■) for 16 weeks, and their body weight was recorded twice a week. **(A)** Changes in body weight are expressed as a gram of measured ± mean body weight weekly. **(B)** Body fat, **(C)** lean body mass; **(D)** total fat; **(E)** fasting blood glucose; and **(F)** parametrial fat were measured at the experimental endpoint at the 16^th^ week before euthanization of mice. The statistical significance between values of each group was assessed by an unpaired Student’s t-test. ^*^p < 0.05 indicate statistically significant differences in body weight, body fat, total fat, fasting blood glucose, and parametrial fat between ND-fed mice and those fed HFD. Values are mean ± SEM; total n = 18 (six mice per group). Data represent the mean of three experiments involving six mice per group with three repeats.

The immune system and metabolic pathways intersect and cross-talk in the adipose tissue microenvironment. This overlap is important for initiating and maintaining adipose tissue (AT) inflammation and can enhance obesity. To determine whether HFD can lead to metabolic changes and obesity, we compared various metabolic markers in mice fed a normal diet (ND) compared with those fed a HFD. As expected, the body weight of mice given HFD increased significantly to 38.2 ± 2.1gm, while those fed a ND weighed 20 ± 1.1gm (**Figure 1A**). Mice fed HFD exhibited statistically significant increases in the percent body fat (**Figure 1B**), lean mass (**Figure 1C**), and total fat (**Figure 1D**), compared to those fed ND. They also showed a significant increase in fasting blood glucose level and parametrial fat (**Figures 1E, F**) compared with the ND-fed controls. These results confirm previous findings that HFD stimulates metabolic markers associated with AT and enhances obesity.

### HFD Induces TCR αβ^+^T Cells Frequency in Epididymal AT

**Figure 2:**
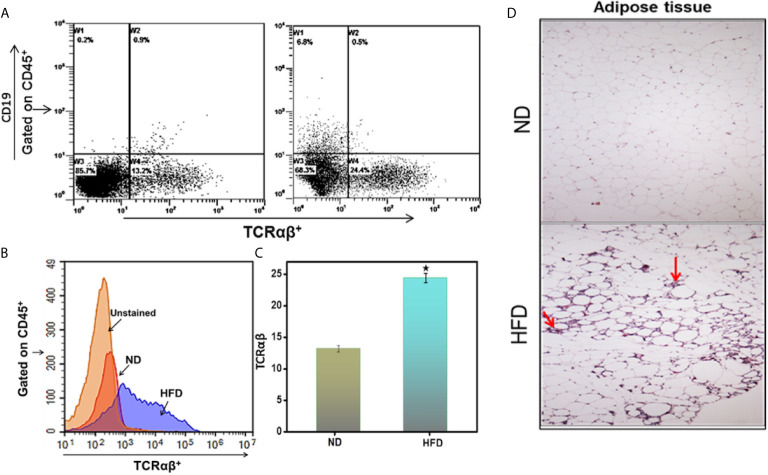
DIO induces immune cell infiltration in the adipose tissue. Peripheral AT was isolated from the two groups of mice (HFD and ND) after 16 weeks and stained for CD45^+^, CD19, and TCRαβ^+^ Abs. **(A)** Shows a representative experiment indicating the percentages of TCRαβ^+^ in the cells gated on CD45^+^ in peripheral fat adipose tissue. **(B)** Shows a representative overlay histogram of TCRαβ^+^. **(C)** Shows cumulative data represent the total percentage of cells ± SEM; total n = 18 (six mice per group) from three independent experiments. The statistical significance between ND and HFD is assessed by Student’s t-test. ^*^p < 0.05 indicate statistically significant differences in TCRαβ^+^ between ND and those fed HFD. **(D)** Shows adipose tissue was isolated and fixed in 4% paraformaldehyde, embedded in paraffin, and sectioned at 6 μm. Sections were stained with hematoxylin and eosin. Sections were examined microscopically at a magnification of 10X by a blinded investigator not related to this study. The mice that received HFD had increased the size of adipose cells and marked leukocyte infiltration (indicated by arrow).

Immune cells, especially T lymphocytes, play essential roles in adaptive immune responses and contribute to obesity-associated inflammation. To determine whether immune cells have infiltrated the epididymal AT after mice are fed HFD, we performed flow cytometry of AT-derived cells. Mice fed HFD exhibited an increased frequency of lymphocyte common antigen-positive (CD45^+^) cells that were positive for T-cell receptor (TCR) αβ, compared to those fed ND (**Figures 2A–C**). These findings indicate that HFD feeding is associated with an increased frequency of TCR αβ^+^T cells in epididymal AT, suggesting the infiltration of T cells into AT. The data at our disposal did not rules out whether limited infiltration of T cells followed subsequent expansion or extensive infiltration in AT that might be responsible for maintaining low-grade chronic inflammation during obesity.

We also performed a histopathological evaluation to confirm these results. The AT of mice fed HFD exhibited increased accumulation of immune cell infiltrates compared to that from ND fed mice (**Figure 2D**, arrow). Adipocyte size also increased in the HFD-fed group as compared to the ND-fed group. These data support our hypothesis that immune cells infiltrate and accumulate in AT of HFD-induced obese mice as compared to ND-fed mice.

### HFD Increases T Cells Frequency in the Spleen, Mesenteric Lymph Nodes, and Epididymal AT

**Figure 3:**
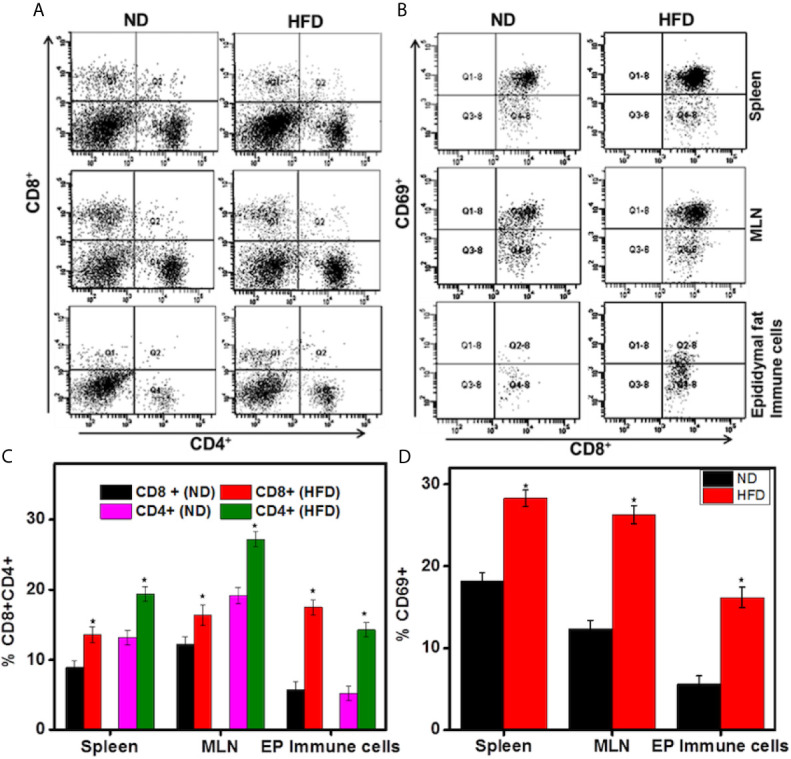
DIO induces activated CD8^+^T cells in the adipose tissue. Changes in CD4^+^, CD8^+^, and CD69^+^ T cells in mice 16 weeks after beginning the HFD. Splenic, mesenteric lymph nodes (MLNs), and epididymal adipose cells were isolated from the two groups of mice. Changes in the frequency and percentage of CD4^+^ and CD8^+^ T cells **(A, C)**, and CD8^+^CD69^+^ T cells **(B, D)** were determined by flow cytometry and expressed as the mean percentage of cells/mouse ± SEM. The statistical significance between ND and HFD is assessed by Student’s t-test. ^*^p < 0.05 indicate statistically significant differences in CD4^+^, CD8^+,^ and CD69^+^ T cells between ND and those fed HFD. The cumulative data shown are from a representative experiment, total n=18; three independent experiments involving six mice per group yielded similar results.

Chronic inflammation and impaired adaptive immune response are hallmarks of obesity (4, 23). Since T cells regulate inflammation in AT (24), we compared the frequency and phenotypes of T cells found in spleens, MLNs, and epididymal AT of mice fed HFD for 16 weeks with those from mice fed ND. The epididymal AT of mice fed HFD contained 2.7-fold more CD4^+^ and 3-fold more CD8^+^ T cells than did that of mice fed ND (**Figure 3A**). Functional characterization of CD8^+^ T cells from each organ like spleen, MLNs, and epididymal AT revealed a striking increase in activated CD8^+^ CD69^+^ T cells in mice fed HFD compared to those given ND (**Figure 3B**). The frequency of CD4^+^, CD8^+^, and CD69^+^ T cells are increased in HFD as compared ND group as shown in the bar graph (**Figures 3C, D**). These findings are consistent with the role of CD8**^+^** T cells in the initiation of inflammation during diet-induced obesity in mice. However, CD8^+^ T cells are prominent during HFD-induced obesity in this study, but we will focus on both subsets of T cells during obesity.

### HFD Increases CD4^+^ CD69^+^ T Cells Frequency and Number in AT

**Figure 4:**
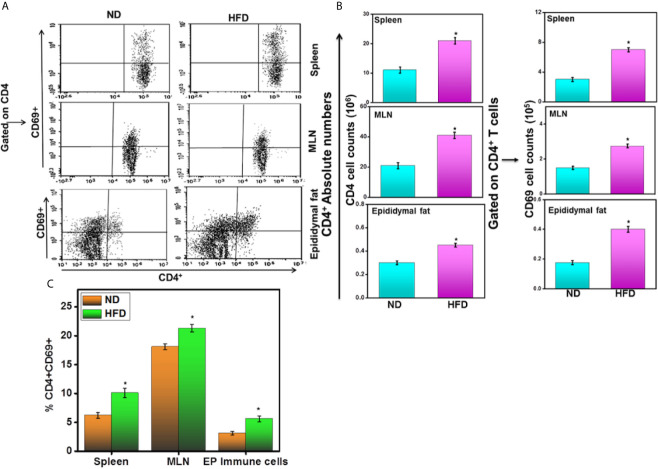
HFD induces activated CD4^+^T cells in the adipose tissue. Changes in CD4^+^ and CD69^+^ T cells in mice 16 weeks after beginning the HFD. Splenic, mesenteric lymph nodes (MLNs), and epididymal adipose cells were isolated from the two groups of mice. Changes in the numbers and percentage of CD4^+^ and CD69^+^ T cells **(A, C)** and CD4^+^ and CD69^+^ T cells number **(B)** were determined by flow cytometry and expressed as the mean percentage of cells/mouse ± SEM; n = 18 (six mice per group). The statistical significance between ND and HFD is assessed by Student’s t-test. ^*^p < 0.05 indicate statistically significant differences in CD4^+^and CD69^+^ T cells between ND and those fed HFD. The cumulative data shown are from a representative experiment; three independent experiments involving six mice per group yielded similar results.

We compared the frequency and number of CD4^+^CD69^+^ T cells found in various systemic and mucosal organs of mice fed HFD for 16 weeks with those from mice fed ND. The spleens, MLNs, and epididymal AT of mice fed HFD contained a significantly higher frequency and number of CD4^+^ T cells as compared with mice fed ND (**Figures 4A, B**). Functional characterization of CD4^+^ T cells from each organ like spleen, MLNs, and epididymal AT revealed a striking increase in activated CD4^+^CD69^+^ T cells in mice fed HFD compared to those given ND (**Figure 4C**). The number of CD4^+^ and CD69^+^ T cells also increased in the spleen, MLNs and epididymal AT of HFD fed mice as compared to ND fed mice (**Figure 4B** right panel). These findings suggest that HFD induced the number and frequency of CD4^+^CD69^+^ T cells that might lead towards Th1 phenotypes response in AT.

### HFD Induces Th1 Phenotypes in AT

**Figure 5:**
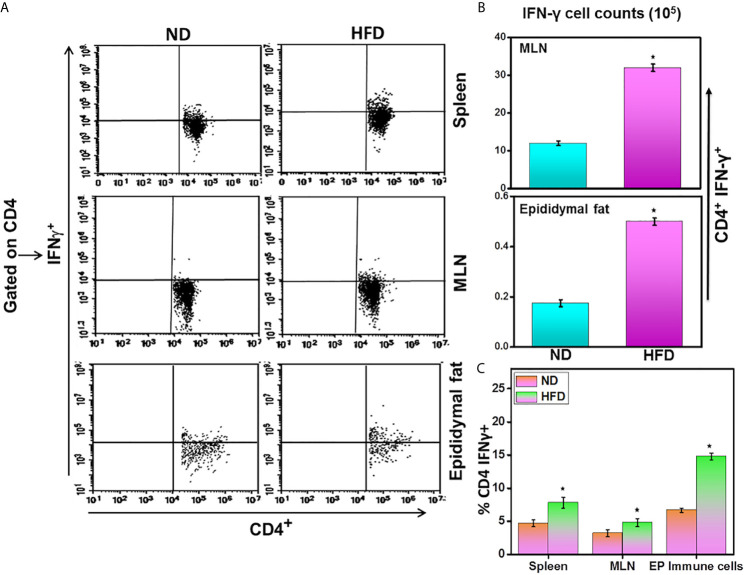
DIO induces Th1 response in adipose tissue. Changes in the absolute numbers and frequency of CD4^+^IFNγ^+^ T cells in mice 16 weeks after beginning the high-fat diet. Splenic, mesenteric lymph nodes (MLNs), and epididymal adipose cells were isolated from the two groups of mice. The cumulative data showed changes in the frequency and numbers of CD4^+^IFNγ^+^ cells **(A, B)**, and the percentage of CD4^+^IFNγ^+^ **(C)** were shown as the mean percentage of cells/mouse ± SEM. Three independent experiments; a total of n = 18 involving six mice per group yielded similar results. ^*^p < 0.05, student’s t-test indicates statistically significant differences in numbers and frequency of CD4^+^IFNγ^+^ in the spleen, MLNs, and epididymal fat between ND and HFD groups.

We compared the number of CD4^+^IFN-γ^+^ T cells found in spleens, MLNs, and epididymal AT of mice fed HFD with those from ND. The epididymal AT, spleen, and MLNs of mice fed HFD contained a significantly higher frequency of IFN-γ^+^ expressing CD4^+^ T cells as compared with ND-fed mice (**Figures 5A, C**). The number of IFN-γ^+^ T cells also increased in MLNs and epididymal AT of HFD fed mice as compared to ND-fed mice (**Figure 5B**). These findings suggest that HFD induces both the number and frequency of CD4^+^IFN-γ^+^ T cells that might lead towards Th1 phenotypes response in adipose tissue.

### HFD-Induced Obesity Mediates the Expression of CXCR3 in AT

**Figure 6:**
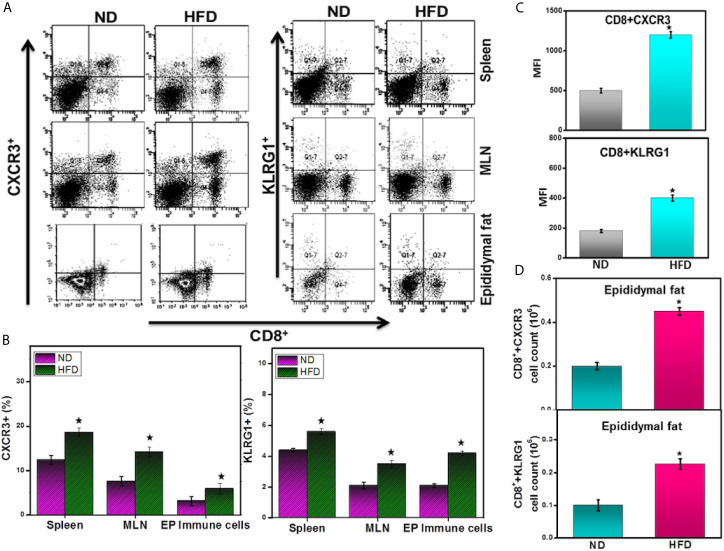
DIO induces the expression of CXCR3 and KLRG1 by CD8^+^ T cells in adipose tissue. In a diet-induced obesity model, HFD mediates CXCR3- and KLRG1-expressing CD8^+^ T cells in mice 16 weeks after induction. Splenic, MLNs and epididymal adipocytes were isolated from the two groups of mice. Changes in the frequency of CD8^+^CXCR3^+^ T cells (**A **left panel and** B**), CD8^+^ KLRG1^+^ T cells (**A **right panel and** B**), changes in CXCR3^+^ and KLRG1^+^ mean fluorescence intensity (MFI) **(C)**, and absolute numbers of CXCR3^+^ and KLRG1^+^ in the AT **(D)** were determined by flow cytometry and expressed as the mean percentage of cells/mice ± SEM. The cumulative data shown are from a total n =18, representative experiment; three independent experiments involving six mice per group yielded similar results. ^*^p < 0.05, unpaired student’s t-test indicates statistically significant differences in MFI and numbers of CXCR3 and KLRG1 between ND and HFD groups.

CXCR3 is required for the maturation of CD8^+^ effector cells and their activation in response to various signals. CXCR3 and its ligands also facilitate T cell differentiation and affect the balance between memory and effector T cells (14, 15). How AT resident cells help to determine the fate of CD8^+^ cell responses and shape populations of memory T cells is unknown. Therefore, we investigated whether HFD induces CXCR3^+^ T cells to initiate an inflammatory response in obese epididymal AT. Mice fed HFD exhibited an increased frequency of CD8^+^CXCR3^+^ T cells in the MLNs and epididymal AT (and a moderate increase in CD4^+^ T cells; data not shown) compared with mice fed ND (**Figures 6A **left panel, **B**). Further, the mean fluorescence intensity (MFI) and absolute number of CD8^+^ CXCR3^+^ T cells are higher in HFD fed mice as compared to ND (**Figures 6C, D**). These data suggest that HFD may mediate the CXCR3 expression to facilitate CD8**^+^** T cells function that regulates adipose inflammation during obesity.

A subset of effector CD8^+^ T cells that co-express the lymphocyte inhibitory receptor KLRG1 and the cytokine IL-7Rα allows investigation of effector cell function, migration, and long-term survival (25). To determine whether diet-induced obesity (DIO) affects KLRG1 expression on CD8^+^ T cells, we performed flow cytometry on immune cells isolated from HFD or ND-fed mice. The mice fed HFD exhibited an increased frequency, number, and mean fluorescence intensity (MFI) of CD8^+^ T cells expressing KLRG1 in MLNs and epididymal AT (**Figures 6A **right panel,** B–D**). These data suggest that the HFD or the DIO that results from it induces KLRG1 expression on CD8**^+^** T cells, most likely to mediate effector function and perhaps inflammation during obesity.

### DIO Mediates the Increase in Macrophage Numbers and Phenotypes

**Figure 7:**
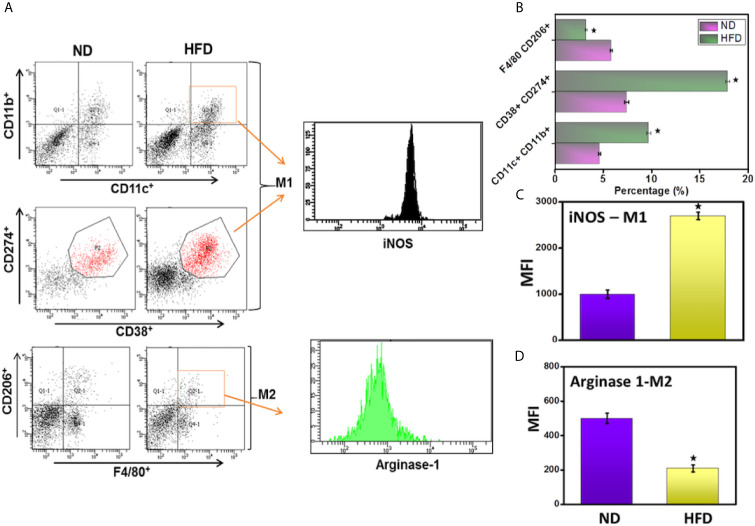
DIO mediates the macrophage frequency and polarization in adipose tissue. The adipocytes were isolated from the two groups of mice after 16 weeks. Changes in the frequency of M1 (iNOS positive cells, upper panel **A**, **B**), M2 (Arginase-1 positive cells, lower panel **A**, **B**), and mean fluorescence intensity (MFI) of both iNOS and Arginase-1 **(C, D)** were determined by flow cytometry and expressed as the mean percentage of cells/mice ± SEM. The cumulative data of dot plot and histogram are shown from a total n=18 representative experiment. The percentage change is shown by combining the mean of the three independent experiments involving six mice per group yielded similar results. ^*^p < 0.05, unpaired student’s t-test indicates statistically significant differences in MFI and frequency of iNOS (M1) and arginase-2 (M2) between ND and HFD groups.

Macrophages protect against harmful local antigens such as those that stimulate inflammation. Macrophages in tissues tend to exhibit either the classical pro-inflammatory M1 phenotype that produces IL-12, IL-23, and inducible nitric oxide synthase (i-NOS), express CD11b, CD11c, CD38, and CD274 cell surface markers and promote a Th1 response or the anti-inflammatory M2 phenotype that produces IL-10 expresses arginase-1 (Arg-1), F4/80, and CD206 cell surface markers, and provides immunoregulatory functions (26, 27). To determine whether HFD induction plays any role in enhancing macrophage number and function, we used flow cytometry to analyze the subsets and phenotypes of systemic and epididymal AT macrophage populations. The number of pro-inflammatory M1 macrophages (CD11b^+^CD11c^+^ and CD38^+^CD274^+^) that also expressed iNOS increased in the AT of mice fed HFD (**Figures 7A **upper panel,** B**). The mice fed HFD also exhibited decreased numbers of F4/80+CD206 (M2) macrophages that expressed Arg-1 (**Figures 7A **lower panel,** B**). Further, this data corresponds with the increased MFI of iNOS and decrease of Arg-1 in the HFD fed group as compared to the ND group (**Figures 7C, D**). This suggests that DIO polarizes macrophages toward pro-inflammatory M1 phenotypes, presumably to induce and maintain chronic inflammation.

### HFD Increases Systemic Inflammatory Chemokines and Cytokines

**Figure 8:**
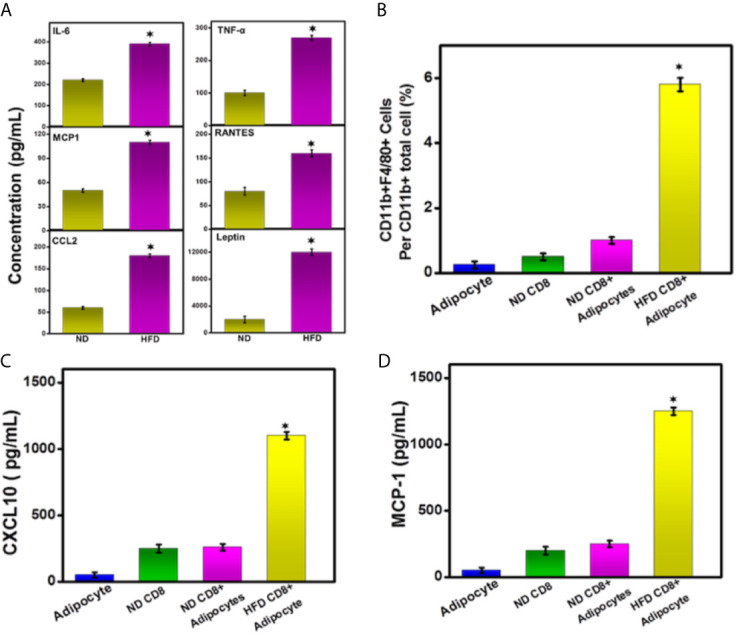
DIO increases the secretion of leptin and systemic cytokines into the serum. HFD induction modulates inflammatory cytokines**. (A)** HFD induces systemic IL-6, MCP-1, IL-1β, TNF-α, RANTES, and leptin at the experimental endpoint after 16 weeks from the groups of mice analyzed by ELISA assay. **(B)** *in vitro* effect of CD8^+^ T cells and adipocyte on differentiation of circulating CD11b^+high^ monocytes. HFD and ND induced CD8^+^ T cells and adipocytes were isolated and purified from epididymal AT. We co-cultured CD8^+^ T cells and adipocytes with circulating monocyte with various combinations. The differentiated macrophage numbers are shown. **(C, D)** Concentrations of CXCL10 and MCP-1 in the culture supernatant are shown. The cumulative data represent the concentration of inflammatory cytokine and chemokines ± SEM from three independent experiments. ^*^p < 0.01, unpaired student’s t-test indicates statistically significant differences in cytokine levels and the number of macrophages between ND and HFD groups.

Obesity and chronic inflammation share common characteristics (28). Obese mice exhibit increased levels of both systemic (IL-1β, IL-6, leptin, MCP-1, RANTES, and TNF-α) and localized AT inflammatory cytokines and chemokines. We used an ELISA assay to measure the levels of these chemokines and cytokines in serum from mice fed HFD or ND. We detected a significant increase in immune and AT-mediated chemokines and cytokines (**Figure 8A**) and a decrease in adiponectin levels (data not shown) in the group of mice fed HFD compared to those fed ND. These data suggest that DIO plays a role in chronic inflammation, at least in part increasing circulating levels of systemic chemokines and cytokines.

### HFD Induced CD8^+^ T Cells Mediates Macrophage Function *via* Cross-Talk With Adipocytes

We next sought to determine whether the HFD induced AT could mediate CD8^+^ T cells function, which could, in turn, enhance the polarization of the macrophage to maintain low-grade inflammation. To test this, we isolated and purified CD8^+^T cells from epididymal AT of mice fed with HFD and ND. Next, we sorted purified circulating peripheral monocytes (CD11b^+^ purity >95%) using a cell sorter (FACS ARIA-II). Further, we also purified adipocytes similarly from HFD and ND-fed mice. We co-cultured adipocyte and CD8^+^ T cells with circulating peripheral monocytes in various combinations for 6 days. Adipocyte and CD8^+^ T cells from ND-fed mice did not induce *in vitro* differentiation to induces macrophage number, and cytokine production (**Figures 8B–D**), while CD8^+^ T cells from HFD fed mice induced an increase in numbers of differentiated macrophage (**Figure 8B**). These data suggest that the DIO-induced CD8^+^T cells were essential for the differentiation of monocytes to macrophages and might be true for *in vivo* conditions to maintain low-grade chronic inflammation typical of obesity. However, we have to do further in-depth studies by using either adoptive transfer or depletion of CD4 and/or CD8 T cells during DIO for a mechanistic and prudent conclusion.

## Discussion

Low-grade chronic inflammation in adipose tissue is a key event leading to metabolic syndrome (MS), type 2 diabetes mellitus (T2DM), and cardiovascular diseases. However, it remains unclear how AT inflammation initiates and mediates infiltrated immune cells to maintain inflammation under obese conditions. Here, we investigated the role of HFD-induced obesity in initiating an immune response in AT and the underlying mechanisms during obesity. We found that activated CD4^+^CD8^+^ T cells infiltrated the AT, leading to the accumulation of M1 macrophages, and observed an increase in the expression of CXCR3 and KLRG1 on CD8^+^ T cells. These data suggest a role of obese AT in both macrophage and CD8^+^ T cells induction and function during obesity. Thus, we believe that a cross-talk between CD8^+^ T cells, CD4^+^ T cells, adipocytes, and macrophages is critically involved in initiating and maintaining inflammation by inducing the production of pro-inflammatory cytokines, chemokines, and leptin in the obese AT microenvironment.

T cells accumulate in obese AT (5, 6). T cells infiltrate the AT early in the development of obesity, before a major influx of macrophages (29). It has been shown that CD8^+^ T cells that have been activated by endogenous stimuli from the adipose microenvironment in the AT have been strongly implicated in the initiation of obesity-associated inflammation (9). Our finding supports the hypothesis that both CD4^+^ and CD8^+^ T cell numbers increase in the epididymal AT due to activation and proliferation. In mice fed an HFD, shown that mainly CD8^+^ T cells express higher levels of CXCR3 and KLRG1, thereby confirming their effector functions, migratory properties, long-term survival, and memory potential (25, 30). Our results suggest that only CD8^+^ T cells induced by feeding of HFD but not ND can induce the macrophages numbers, polarization, and production of cytokines characteristic of obesity lend credence to this hypothesis. This data further suggests the existence of a key interaction between a stimulus from HFD-induced AT and the CD8^+^ T cells and macrophages that infiltrate it, leading to the production of cytokines and chemokines to propagate local tissue inflammation. This is in good agreement and supports the earlier study and mechanistic evidence by Nishimura et al. (9). Further, the addition of HFD also significantly changes the number of CD4^+^CD69^+^ T cells, as well as induces IFN-γ response which might also have been expected to contribute to local inflammation within the AT microenvironment. In contrast, it has been shown that subsets of CD4^+^ T cells can secrete IL-10 and regulatory T cells (Tregs) can suppress T cell responses (31, 32). However, experiments on HFD mediated Tregs frequency and function in AT are beyond the scope of this study.

CXCR3 receptors and their ligands CXCL9 and CXCL10 control T cell trafficking and are important for the migratory behavior and function of CD8^+^ effector and memory T cells (33, 34). The activated CD8^+^ T lymphocytes that express high levels of CXCR3 receptors (and their ligands CXCL9 and CXCL10) primarily attract activated CD4^+^ T helper type 1 (Th1) T cells (18, 35, 36). Adipocytes also express CXCR3 receptors and modulate obesity-induced inflammation (19). A clue to the possible mechanism by which this occurs came from the observation that CXCR3 receptors facilitate differentiation of CD8^+^ T cells into short-lived effector cells, leading to a degeneration of T cell memory and thereby affecting the balance between the production of memory and effector CD8^+^ T cells (14, 15). Macrophages also express CXCR3 receptors, which may play a role in their activation (37). Our observation of a statistically significant increase in CD8^+^ T cells positive for CXCR3 and KLRG1 in adipose tissue after HFD induction lends credence to the suggestion that CXCR3 induces CD8^+^ T cells activation, migration, and production of inflammatory cytokines (CXCL10 and MCP-1). CXCR3 expression may also assist in the production of a hypothetical long-term memory cell in obese microenvironments that serves to sustain low-grade inflammation in AT.

In addition to their roles as antigen-presenting cells, macrophages are also an essential component of the innate immune system that can change their status to support immune responses or the development of obesity and related metabolic diseases. In both mice and humans, AT is infiltrated by macrophages (4) and effector T cells (T_effs_) recruit macrophages during AT inflammation (9). In obesity, the numbers of M1 macrophages present in AT increase, which correlates with AT inflammation and insulin resistance (13). In the present study, we observed an increase in M1 macrophages and a decrease in M2 macrophages in HFD that corroborates the previous findings by others. The results from the co-culture experiment we performed, shows that monocyte differentiation to macrophage and phenotypic polarization are mediated in part by the interaction of HFD induced CD8^+^ T cells with adipocytes, which may serve to regulate the local inflammatory cascade. This is further confirmed by our observation that neither ND induced CD8^+^ T cells alone, nor adipocytes from ND mice were sufficient to induce macrophage numbers. Thus, the results of this study strongly suggest the existence of a cross-talk between adipocytes, CD8^+^ T cells, and macrophages in an obese AT environment that propagates local adipose inflammation.

Feeding mice with HFD leads to the increased secretion of pro-inflammatory cytokines from adipose macrophages, T cells, and adipocytes, which contribute to obesity-associated inflammation. During obesity, macrophages switch to an activated state and secrete MCP-1 and TNF-α. Obesity is also associated with an elevated level of pro-inflammatory cytokines and chemokines IL-6, RANTES, and IL-1β and of the hormone leptin, which maintains homeostatic control of AT mass (38, 39). In this study, we observed an increase in systemic concentrations of IL-6, RANTES, IL-1β, MCP-1, and leptin in the serum of HFD fed mice, as compared to the levels observed with mice fed ND. These data corroborate with previous studies and suggest the hypothesis that during obesity, activated M1 macrophages induce a Th1 response in the AT microenvironment and increase the production of pro-inflammatory cytokine and chemokines (40, 41). We also observed an increase in cytokines, chemokines, and the CXCR3 ligand CXCL10 in the co-culture supernatant of HFD-induced CD8^+^ T cells, macrophages, and adipocytes compared to the levels obtained from ND fed mice. We hypothesize that induction by HFD leads to the induction of CD8^+^ T cells in AT and polarization towards M1 macrophages that secrete MCP-1 and CXCL10 to maintain low-grade chronic inflammation, as described in a previous study (42).

In summary, our results support the hypothesis that obese AT induced by HFD activates both CD4^+^ CD8^+^ T cells, which initiate and propagate an inflammatory cascade by recruiting macrophages to obese adipose tissue, where they further differentiate into M1 macrophages. It is most likely the case that infiltrated CD8^+^ T cells maintain their activation function through increased expression of CXCR3 and KLRG1 in obese AT. Further, infiltrated activated CD4^+^ T cells induce IFN-γ response to maintain Th1 response and low-grade adipose inflammation. These combined effects are likely to be responsible for the continuous differentiation of macrophages to a pro-inflammatory M1 phenotype that secretes cytokines and chemokines to sustain low-grade chronic inflammation in obese tissue. However, a more detailed study will be required before any firm conclusions may be drawn from the other environmental cues within obese AT that may initiate the recruitment and or expansion of CD8^+^ T cells. Our results suggest that AT inflammation has a major impact on systemic metabolism as well as being the root cause of many autoimmune diseases and cancer.

## Data Availability Statement

The raw data supporting the conclusions of this article will be made available by the authors, without undue reservation.

## Ethics Statement

All animal experimentation was performed under protocols approved by the University of South Carolina’s and University of Tennessee Health Science Center’s Institutional Animal Care and Use Committee (IACUC) and handling and experimental procedures were performed to minimize pain and discomfort.

## Author Contributions

US conceived the ideas, wrote the manuscript, and performed the work and data analysis. EM helped with data analysis and edited the manuscript. RE performed part of the work at University of South Carolina. SK and VK performed part of the work, made the figures, was involved in data analysis, and assisted in the writing of the manuscript. All authors contributed to the article and approved the submitted version.

## Funding

This study was supported in part by grants from NIAID R01 AI140405 to US, and the Intramural Research Program at UTHSC in Memphis, TN.

## Conflict of Interest

The authors declare that the research was conducted in the absence of any commercial or financial relationships that could be construed as a potential conflict of interest.
